# Nerve Blockage Attenuates Postoperative Inflammation in Hippocampus of Young Rat Model with Surgical Trauma

**DOI:** 10.1155/2015/460125

**Published:** 2015-11-19

**Authors:** Yi He, Zhi Li, Yun-Xia Zuo

**Affiliations:** ^1^Department of Anesthesiology and Translational Neuroscience Center, West China Hospital, Sichuan University, Chengdu, Sichuan 610041, China; ^2^Department of Critical Medicine, Chengdu Shang Jin Nan Fu Hospital, Sichuan 610041, China

## Abstract

It is hypothesized that central nervous system inflammation induced by systematic inflammation due to surgical trauma plays a critical role in postoperative cognitive dysfunction. The potential inhibitory effect of nerve blockage with local anesthetics on peripheral inflammatory response has been reported. We hypothesize that nerve blockage may be effective in reducing postoperative inflammation and cognitive decline. The rats at the age of 4 weeks were subjected to general anesthesia and humeral fracture fixation, in combination with brachial plexus block, saline versus ropivacaine, respectively. The rats from control group underwent general anesthesia only. The expression of proinflammatory cytokines in plasma and in hippocampus was measured. Open field test and new object recognition task were performed before surgery and on postoperative days (POD) 1, 3, and 7. Compared with control group, the level of cytokines in plasma and hippocampus revealed an obvious increase in surgery groups. The effect of brachial plexus block on decreasing cytokines was observed. The rats exposed to surgery without brachial plexus block showed behavior impairment. Our results indicated that nerve blockage could downregulate proinflammatory cytokines in hippocampus after humeral fixation surgery, which may ameliorate the postoperative cognitive dysfunction in young rats.

## 1. Introduction

Accumulated evidence indicated that surgery-induced central nervous system (CNS) inflammation plays a key role in postoperative-deteriorated cognitive function [1–7]. Inflammatory response of CNS is a part of systemic inflammation. Surgical trauma can initiate peripheral immune activation, thereby leading to systemic cascade responses of proinflammatory cytokines [8–10]. The peripherally originating proinflammatory cytokines may enter into CNS through a direct or indirect way [9, 11]. Excessive inflammatory mediators in brain can influence postoperative learning development. Therefore, if the responses triggered by surgical noxious stimuli were inhibited, CNS inflammation may be attenuated and postoperative cognitive dysfunction can be mitigated.

Peripheral nerve blockage may be an effective strategy to reduce the noxious stimuli, thus resulting in the attenuation of inflammation caused by surgery in the extremities. Local anesthetics (LAs) have been demonstrated to exert the influence on modulating peripheral inflammatory responses and reducing memory impairment [12, 13]. LAs can inhibit axonal transport and liberation of several proinflammatory cytokines, such as interleukin-1*β* (IL-1*β*), interleukin-6 (IL-6), and tumor necrosis factor-*α* (TNF-*α*) [11, 13–15]. Thus, we hypothesize that peripheral nerve blockage may be an effective strategy to reduce postoperative inflammation, thereby mitigating postoperative learning impairment. In the present study, young rat model with humeral fracture and reduced fixation was established. The effects of brachial plexus block on open field test and new object recognition task as well as plasma/hippocampus levels of proinflammatory cytokines were investigated.

## 2. Methods

### 2.1. Animals and Experimental Grouping

The 4-week male Sprague-Dawley rats (body weight, 90 ± 10 g) were housed in groups of 6 under the environment with controlled temperature, 12 h light/dark cycle, and free accessibility to food and water. Experimental protocols were approved by the Scientific and Ethics Committee of West China Hospital, Sichuan University, and conformed to National Institutes of Health guideline. Animals were randomly assigned into three groups. Rats in the control group (group C, *n* = 36) only received general anesthesia. The rats from the other two groups received general anesthesia and humeral fracture fixation surgery, in combination with brachial plexus block, saline (group S, *n* = 36) versus 0.5% ropivacaine (group BPB, *n* = 36) at the volume of 0.2 mL, respectively [16–18].

### 2.2. Humeral Fracture Fixation Surgery and Brachial Plexus Block

Rats without any signs of preprocedural neurobehavioral impairments were anesthetized with a combination of 2-3% sevoflurane with 50% oxygen and 10 *μ*g/kg fentanyl (subcutaneous injection). The left brachial plexus was exposed through a 1 cm infraclavicular incision, and then blunt dissection of the muscle was conducted to reveal the subclavian artery. Following the dissection, the brachial plexus were clearly identified near the artery. All nerve blockage was performed under direct vision using a tuberculin syringe to inject ropivacaine into the perineural space below the fascia covering the nerve. Then, the left upper limb of the animal receiving humeral fracture fixation surgery was sterilized. A longitudinal incision of 0.5 cm was made from the lateral to left humerus and then the muscle fascia was slightly dissected till the observation of the bone. With ophthalmic scissors, an osteotomy in the middle of left humerus was performed. A humeral fracture fixation was followed. Briefly, a skin incision ran along the outer edge of the acromion; the deltoid muscle was exposed. With identifying the cephalic vein, a 0.88 mm Kirschner wire was drilled into medullary canal from the proximal end to the distal end of the humerus under direct vision. The Kirschner wire was cut flush with the humeral cortex and the skin closed directly with 6-0 Ethicon sutures. Rats were recovered after intervention and then returned to their cages. The entire surgical procedure took 10–15 minutes.

### 2.3. Behavior Tests

Rats were accustomed to handling for at least 3 min daily for 3 days prior to the experiment. This handling procedure was aimed at reducing possible stress. An hour before the test, the cages with the rats were brought to testing room adjacent to the environment. Behavior tests including open field test and new object recognition task (familiarize with the testing environment) were performed prior to the surgery and at postoperative days (POD) 1, 3, and 7.

#### 2.3.1. Open Field Test

It is widely used to assess locomotor, exploratory, and anxiety-like behavior in rodents. A tested rat was removed in a random order from the cage to the open field (a 142 × 142 × 30 cm box illuminated in the center of the arena by a 20 W white light suspended from a floor lamp), gently released into the center of the apparatus, and allowed to explore the area for 5 minutes. Data including total distance moved and time spent in the inner area were analyzed.

#### 2.3.2. New Object Recognition Task

The new object recognition test was performed according to the protocol [19]. Twenty-four hours before the test, rats were put in the apparatus for object recognition task (78 cm × 56 cm × 43 cm) for 20 min separately to familiarize with the testing environment so that environmental exploration does not interfere with object interactions. The next day, two identical objects (bowls or square one) were put in the box. Then each rat was placed individually at the midpoint as well as the frontal wall of the box, opposite the objects. They were allowed to explore objects for 10 min and then were taken back to the cages (familiarization phase). An hour later, one of the familiar objects was replaced by a novel object, and rats were returned to the apparatus with the same object and a novel object to explore for 5 min (testing phase). All processes were videotaped. Directed contacts scoring including any contact with mouth, nose, or paw with each object are manually calculated by the experimenter and an investigator who was blind to the experimental design. Basic measurements include exploratory time with familiar object versus novel object and a discrimination ratio (novel object interaction/total interaction with both objects) [19].

### 2.4. Surgery-Induced Inflammatory Response

Rats in each group were euthanized on POD 1, 3, and 7 (*n* = 12/time point) with an overdose of pentobarbital sodium (100 mg/kg, intraperitoneal) after behavior tests. Blood was quickly withdrawn through the abdominal aorta and then centrifuged (3,000 rpm, 10 min, 4°C). Plasma was collected and stored at −80°C until assays. Plasma cytokines (TNF-*α*, IL-6, and IL-1*β*) were assayed (*n* = 6/time point) using ELISA kits (RayBiotech, USA) according to the manufacturer's protocol. All samples were determined in duplicate. The average absorbance of each set of duplicate standards and control samples was calculated. Then, the hippocampus of half the rats (*n* = 6/time point) was quickly dissected, placed into a 1.5 mL tube, and stored at −80°C for cytokine mRNA test. RNA was extracted from homogenization of hippocampal tissue in Trizol reagent (TaKaRa, Japan) in accordance with the manufacturer's manual. Total RNA concentrations were measured with a spectrophotometry at 260 nm and 280 nm (Nanodrop 2000/2000c, Thermo). The M-MLV reverse transcriptase (Promega, USA) was used to reverse-transcribe RNA into complementary DNA (cDNA). The obtained cDNA served as a template for the real-time PCR using 10 *μ*L SYBR premix ex taq (TAKARA, Japan), 8 *μ*L RNase-free H_2_O, 0.5 *μ*L forward primers (2.5 *μ*M), and 0.5 *μ*L reverse primers (2.5 *μ*M) of IL-1*β*, IL-6, TNF-*α*, and GAPDH (Table 1), respectively. The real-time PCR reaction was performed using Thermal Cycler Dice real-time PCT system (TP800, TaKaRa Bio). The expression of IL-1*β*, IL-6, and TNF-*α* was determined using the comparative threshold cycle (Ct) method and normalized by the copy number of GAPDH. The results were expressed as fold change relative to the control group.

The rest of the rats (*n* = 6/time point) were transcardially perfused with 4% paraformaldehyde (PA) in 0.5 M phosphate-buffered saline (PBS). The brain tissues of perfused rats were taken and kept in 4% PA and then embedded in paraffin before being sectioned. Sections were deparaffinized, rehydrated, and soaked in 0.01 M citrate buffer (pH 6.0) for 3 min high pressure repair and cooled at room temperature before being washed with PBS for 3 times. After preincubation 20 min with 0.3% hydrogen peroxide in methanol, the sections were rinsed three times with PBS and then incubated with normal goat serum for 10–15 min at room temperature. The rabbit anti-S100*β* monoclonal antibody (1 : 250; Epitomics, USA) and the biotinylated goat anti-rabbit IgG were used as the primary and secondary antibodies. The DAB method was used to visualize reaction products. The sections were visualized at 400x magnification and using the Image-Pro Plus 6.0 to estimate Integrated OD average.

### 2.5. Statistical Analysis

Data are showed as mean ± SD. Normality was tested with the Kolmogorov-Smirnov normality test. Equality of variances was tested with *F* test. The data of blood concentrations and mRNA expression were fitted by a log transformation to better satisfy with the prerequisite assumptions of normally distributed residuals and homogeneity of variance. Mean concentrations of cytokines were evaluated by one-way ANOVA test followed by LSD post hoc test. Nonparametric data were analyzed by Kruskal-Wallis test. A significant difference was considered at *P* value less than 0.05. SPSS was used to conduct the statistical analysis.

## 3. Results

### 3.1. Plasma Inflammatory Cytokine Level and CNS Inflammation

**Figure 1:**
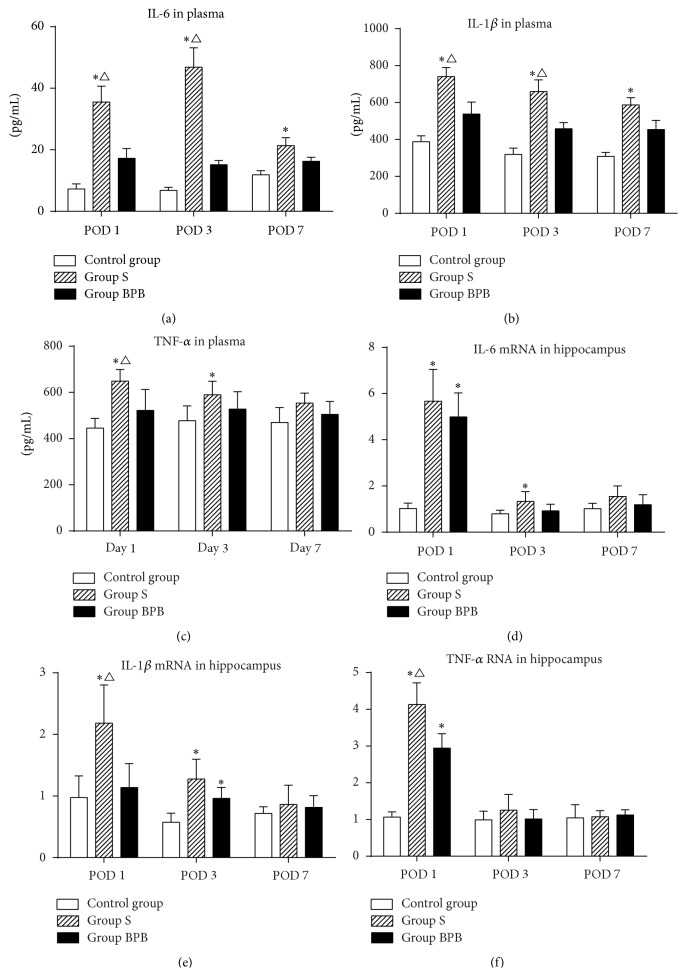
The levels of proinflammatory cytokines in plasma and in hippocampus. Each bar represents mean ± SEM (*n* = 6 in each group). ^*∗*^ *P* < 0.05 versus control group, ^∆^ *P* < 0.05 versus group BPB. POD = postoperative day.

In order to explore whether surgical injury and nerve blockage therapy can alter inflammatory cytokines in blood, plasma level of IL-1*β*, IL-6, and TNF-*α* was measured. Compared with the control group, the plasma levels of cytokines in rats that underwent brachial plexus block before surgery (group BPB) did not exhibit a significant increase (Figure 1, *P* > 0.05) at any test time after surgery; however, the levels of cytokines in rats following humeral fracture fixation without nerve blockage (group S) present an obvious increase on POD 1 (Figure 1, IL-1*β*: *P* = 0.001; IL-6/TNF-*α*: *P* = 0.000), POD 3 (Figure 1, IL-1*β*/IL-6: *P* = 0.000; TNF-*α*: *P* = 0.032), and POD 7 (Figure 1, IL-1*β*: *P* = 0.001; IL-6: *P* = 0.009). Compared with the group BPB, the group S had higher expression level of IL-1*β*/IL-6 on POD 1 (Figure 1, IL-1*β*: *P* = 0.039; IL-6: *P* = 0.008) and POD 3 (Figure 1, IL-1*β*: *P* = 0.022; IL-6: *P* = 0.000) and higher TNF-*α* level on POD 1 (Figure 1, *P* = 0.012).

In order to explore whether surgical injury and nerve blockage therapy can change cytokine expression in brain, the levels of IL-1*β*, IL-6, and TNF-*α* mRNA in hippocampus were measured. Compared with the control group, the elevation of TNF-*α*/IL-6 mRNA in both group BPB (Figure 1, IL-6/TNF-*α*: *P* = 0.000) and group S (Figure 1, IL-1*β*: *P* = 0.004; IL-6/TNF-*α*: *P* = 0.000) was noted on POD 1; however, the upregulation trend was only detected in group S on POD 3 (Figure 1, IL-1*β*: *P* = 0.000; IL-6: *P* = 0.026). Compared with the group BPB, the expression of IL-1*β*/TNF-*α* mRNA in group S was higher on POD 1 (Figure 1, IL-1*β*: *P* = 0.008; TNF-*α*: *P* = 0.001).

**Figure 2:**
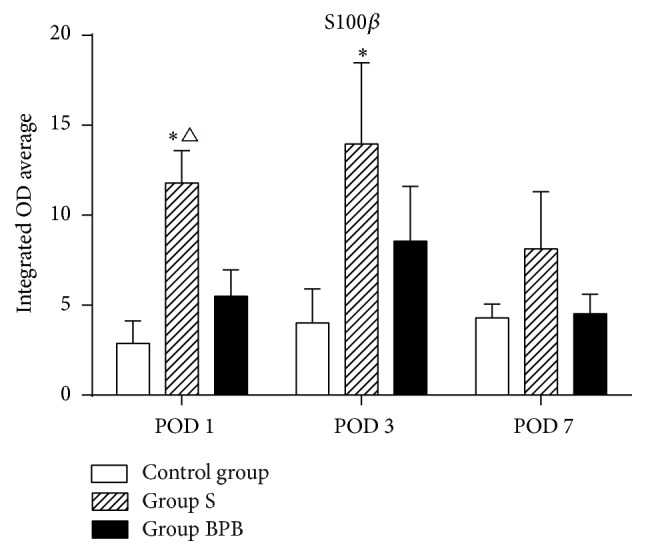
The average integrated OD of S100*β* in hippocampus. Each bar represents mean ± SEM (*n* = 6 in each group). ^*∗*^ *P* < 0.05 versus control group and ^∆^ *P* < 0.05 versus group BPB. POD = postoperative day.

Many previous studies have revealed that S100*β* can activate microglia and plays an important role in neuroinflammation [20]. In order to investigate why such difference in the expression of inflammatory cytokines after surgery was present, hippocampal S100*β* was examined. Compared to the control group, no obvious increase of S100*β* expression in hippocampus of the rats from group BPB was observed (Figure 2, *P* > 0.05); however, the expression level of S100*β* in group S increased on POD 1 and 3 (*P* = 0.000/0.005). A significant difference in S100*β* expressions between group S and group BPB was observed only on POD 1 (*P* = 0.001).

### 3.2. Behavior Change after Humeral Fracture Fixation Surgery

Before surgery, no significant difference in travel distance and time spent in the center was observed among three groups in open field test. On POD 1, compared to the rats from the control group, the rats in group S moved less distance (*P* = 0.001); however, no difference in distance moved in group BPB was shown (*P* > 0.05). There is also statistically significant difference in traveling distance between the group S and group BPB observed on POD 1 (*P* = 0.011). The time spent in the center zone was not different at any measuring time points among three groups (*P* > 0.05).

**Figure 3:**
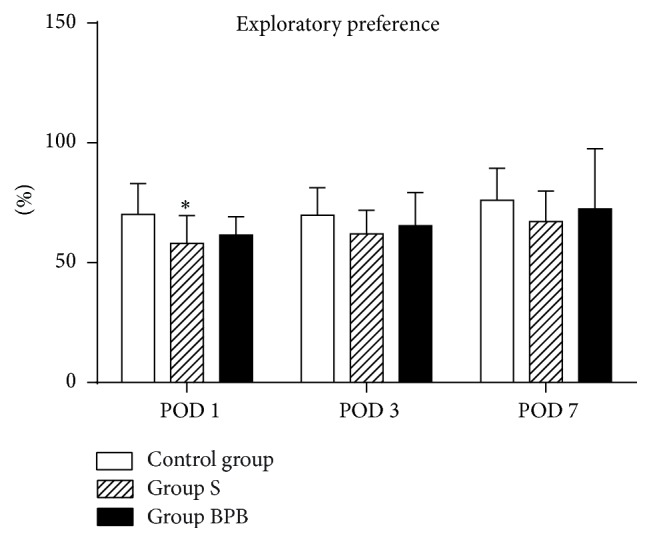
Effect of brachial plexus block on recognition memory assessed by the novel object recognition test. Each bar represents mean ± SEM (*n* = 12 in each group). ^*∗*^ *P* < 0.05 versus control group. POD = postoperative day.

In new object recognition test, the statistically significant difference in the discrimination ratio between control group and group S was noted on POD 1 (Figure 3, *P* = 0.045). No significant difference was observed between group S and group BPB on any time after surgery. Total exploration times during testing phase in group S tended to be shorter than that in group BPB and control group, but the difference was not of statistical significance (*P* > 0.05, Table 2).

## 4. Discussion

In our experiment, young rats from fracture group without performing brachial plexus block revealed an upregulated release of plasma/hippocampal proinflammatory cytokines and suffered postoperative cognitive impairment. However, such exaggerated release of cytokines could be attenuated by brachial plexus block.

Surgical injury can activate peripheral innate immune, which has been reported to cause proinflammatory cytokines in central nervous system, thus contributing to the development of postoperative cognitive decline. Previous studies have shown that different proinflammatory cytokines, especially IL-1*β*, IL-6, and TNF-*α*, are involved in the development of surgery-induced CNS inflammation and cognitive dysfunction [1, 4–10]. In addition, proinflammatory cytokine receptor antagonists or antibodies are demonstrated to be effective in preventing postoperative cognitive dysfunction [2, 8, 9]. Taking comprehensive consideration, surgery-induced CNS inflammation is likely to be one of the mechanisms of postoperative cognitive impairment, and therefore, intervention aimed at reversing the inflammatory processes secondary to surgery injury could be a possible therapeutic target for the postsurgical cognitive impairment.

In the periphery, substance P (SP), calcitonin gene-related peptide (CGRP), and vasoactive peptides were secreted by nerve ending of small fibers following injuries. Anterograde transport of CGRP and SP induces neurogenic inflammation and the secretion of vasoactive peptides resulted from macrophage infiltration, lymphocyte activation, and rapid accumulation of proinflammatory cytokines at the peripheral site of injury, thereby evoking a cascade of inflammatory responses [21]. The cytokines originating from periphery may directly enter into CNS through the relatively permeable blood-brain barrier or bind to its cognate receptors on endothelial cells within brain or through neural afferents [9]. Indirectly, through the vagal afferent nerves, the cytokines can induce the changes within CNS. Retrograde transport of SP and CGRP leads to neurogenic inflammatory response in periphery and CNS. Besides, proinflammatory cytokines triggered by inflammatory insult retrograde transport along small fiber axons to the dorsal root ganglion and spinal cord, thus participating in CNS inflammation [11]. The above-mentioned events initiated in the periphery can activate glial cells within central nervous system. Activated microglia, resident immune cells in brain, accompanied with the elevation of proinflammatory cytokines in brain contribute to the alterations in postoperative cognitive capacity [22]. Nerve blockage has been reported to decrease the neurogenic inflammation. Local anesthetics have anti-inflammatory properties independent of their nerve blocking effects. Recent studies have also reported that nerve blockage can inhibit the axonal transport and then limit the local release of SP and control postoperative analgesia, which may reduce the neurogenic inflammation resulting from peripheral injury [11]. Our results indicated peripheral nerve blockage could attenuate both systemic and central inflammation after orthopedic surgery and therefore provide a potential strategy to prevent postoperative cognitive dysfunction.

Previous studies have suggested that the incidence of postoperative learning dysfunction after minor surgeries was much lower than that in other major surgeries (7–26%) [4]. It has been demonstrated that the concentrations of cytokines in hippocampus revealed an obvious increase after a minor abdominal surgery for 24 hours but did not lead to cognitive deficit during Morris water maze test [23]. In our experiment, the recognition memory deficit was only detected on POD 1. These findings may speculate that the extent of surgery could attribute to postoperative cognitive impairment. More severity of surgical trauma can result in the higher magnitude of postoperative inflammatory response and larger cognitive impairment. The multiple fracture trauma rat models may be used in further researches.

Neurodevelopment impairment and subsequent cognitive learning decline are observed in children after surgery have been increasingly reported [24]. The safety of pediatric surgery and the possible intervention to avoid postoperative neurodevelopment impairment have become major health issues recently. For the past few years, with the spread of ultrasound technology, peripheral nerve blocks are emerging as a safe and effective technique in children [25]. Local anesthetics have also been found to exert significant impact on several other settings, including reducing memory impairment [13] and inhibiting systemic inflammatory responses [12, 15]. Therefore, we established a fracture model in young rats mimicking young/adolescent patients undergoing orthopedic surgery. Together with our findings, nerve blockage therapy may be a potential intervention for anti-inflammatory pathway to improve postoperative cognitive impairment after pediatric orthopedic surgery. However, animal models cannot completely reproduce clinical situations. Whether similar changes occur in pediatric patients needs to be further defined in studies.

In our study, the sevoflurane-anesthetized rats without fracture surgery were used as the control group. However, according to previous suggestions, the inhalation anesthesia exposure also could impair the cognitive function [24, 26, 27]. Further studies need to include a control group receiving neither surgery nor anesthesia to assess the potential influence of anesthesia.

In conclusion, nerve blockage could downregulate proinflammatory cytokines in hippocampus after humeral fixation surgery, which may improve the postoperative cognitive dysfunction in young rats. The potential strategies to alleviate the inflammatory response following surgical injury may attenuate the progress of postsurgical cognitive impairment.

## Figures and Tables

**Table 1:** Primers of proinflammatory cytokines.

Gene	Primer sequence (5′ → 3′)	Amplification size (bp)
IL-1*β*	F-GACAAGCAACGACAAAATCCC	154
	R-GAAGACAAACCGCTTTTCCATC	

IL-6	F-CTTCCAGCCAGTTGCCTTC	107
	R-GTCTGTTGTGGGTGGTATCC	

TNF-*α*	F-GCGTGTTCATCCGTTCT	201
	R-GCCACTACTTCAGCGTCTC	

GAPDH	F-TTCAACGGCACAGTCAAGG	114
	R-CTCAGCACCAGCATCACC	

F: forward primer; R: reverse primer.

**Table 2:** Total time spent exploring the two objects during testing phase.

	Control group	Group S	Group BPB
POD 1	30.63 ± 7.03	26.29 ± 3.47	28.79 ± 3.63
POD 3	28.36 ± 4.77	26.67 ± 4.13	28.63 ± 4.73
POD 7	29.31 ± 4.25	27.54 ± 3.04	29.58 ± 6.25

Total exploration time during testing phase is expressed as mean ± SD in seconds. Each group consisted of 12 animals (*n* = 12 in each group).

POD = postoperative day.
